# Wnt1在非小细胞肺癌中的表达与预后的关系

**DOI:** 10.3779/j.issn.1009-3419.2010.06.004

**Published:** 2010-06-20

**Authors:** 琴 王, 平 展, 力克 于, 勇 宋

**Affiliations:** 1 210002 南京，解放军第八一医院呼吸科 Department of Respiratory Medicine, Nanjing 81 Hospital of PLA, Nanjing 210002, China; 2 210019 南京，南京市胸科医院呼吸科 Department of Respiratory Medicine, Nanjing Chest Hospital, Nanjing 210019, China; 3 210002 南京，南京军区 南京总医院呼吸科 Department of Respiratory Medicine, Nanjing General Hospital of Nanjing Military Command, Nanjing 210002, China

**Keywords:** Wnt1, 肺肿瘤, 免疫组化, 预后, Wnt1, Lung neoplasms, IHC, prognosis

## Abstract

**背景与目的:**

Wnt1蛋白是Wnt信号传导通路的第一个因子，其高表达与很多肿瘤相关，本研究旨在探讨Wnt1蛋白在非小细胞肺癌组织（non-small cell lung cancer, NSCLC）中的表达及与预后的相关性。

**方法:**

选取术后经病理证实的115例NSCLC和19例肺良性病变（5例肺结核、4例支气管扩张、6例肺大疱、4例炎性假瘤），运用免疫组化*Envision*法检测Wnt1蛋白的表达，采用χ^2^检验分析Wnt1蛋白在NSCLC及良性组织中表达的差异及NSCLC中Wnt1蛋白表达与临床病理特征的相关性。采用*Kaplan-Meier*生存分析和*Cox*回归分析方法分析Wnt1蛋白在NSCLC组织中的表达和预后的关系。

**结果:**

Wnt1在NSCLC组织中表达的阳性率为62.6%，显著高于对照组的31.6%（χ^2^=4.474, *P*=0.034），但与临床病理特征无相关性。*Kaplan-Meier*生存分析、*Log-rank*检验提示Wnt1阳性表达的NSCLC的患者预后较差（*P*=0.003），*Cox*回归分析结果表明，Wnt1蛋白是影响NSCLC预后的独立危险因素（OR=1.834, *P*=0.032）。

**结论:**

Wnt1蛋白在NSCLC组织中的阳性表达率高于肺良性病变组织；Wnt1蛋白阳性表达者预后差，可以作为判断NSCLC预后的参考指标。

## Abstract (en)

**Background and objective:**

Wnt1 protein is the first factor in Wnt signaling pathway. It has been proven that high expression of Wnt1 protein was associated with malignant proliferation in many tumors. The aim of this study is to investigate the correlation between Wnt1 protein overexpression, clinicopathologic features and survival in resected non-smallcell lung cancer (NSCLC).

**Methods:**

Immunohistochemical staining of Envision was applied to investigate the expression of Wnt1 protein in specimens of 115 NSCLC and 19 benign pulmonary diseases. The correlation between the Wnt1 protein in specimens of 115 NSCLC and clinicopathologic features was analysed with χ^2^ test, and the correlation between the Wnt1 protein expression and the patient survival was evaluated with *Kaplan-Meier* survival curve and *Cox* regression.

**Results:**

The positive rate of Wnt1 protein in specimens of NSCLC was 62.6%, which was significantly higher than 31.6% of benign pulmonary diseases (χ^2^=4.474, *P*=0.034). But it was not correlated with clinicopathologic features. *Kaplan-Meier* survival analysis and *Log-rank* test suggested that patients with Wnt1 protein overexpression had poor prognosis (*P*=0.003). And *Cox* regression suggested Wnt1 protein expression was an independent prognostic factor of NSCLC.

**Conclusion:**

The expression of Wnt1 was remarkably higher in specimens of resected NSCLC than that in benign pulmonary diseases. Overexpression of Wnt1 protein in NSCLC was correlated with prognosis and can be served as an independent prognostic factor of NSCLC.

肺癌作为一种恶性肿瘤，其发病率和死亡率近年来一直呈上升趋势，已成为21世纪全球面临的严峻的健康问题^[1]^。在中国，据预测到2025年，肺癌患者将达到100万，居世界第1位。肺癌的发病机制复杂，是多因素、多基因、多阶段共同参与的结果，目前这方面的研究主要集中在肿瘤相关基因的克隆和功能分析、细胞信号转导途径及细胞周期调控三大领域，其中细胞信号转导途径对其发生发展起着至关重要的作用。Wnt信号通路是一种进化上高度保守的、对控制胚胎发育有重要作用的信号转导通路。对*Wnt*基因家族成员的编码产物及其生物学效应的研究发现，Wnt信号通路的异常激活参与了多种人类癌症的发生^[2]^。Wnt1蛋白是Wnt通路中第一个被发现的蛋白^[3]^，目前关于Wnt1在非小细胞肺癌(non-small cell lung cancer, NSCLC)中的表达及与术后生存期的相关性研究尚少，为此我们探讨Wnt1在NSCLC组织内的表达情况及其临床意义。

## 1 材料与方法

### 1.1 病人资料

选取解放军第八一医院和南京市胸科医院2001年1月-2005年12月手术切除的经常规病理证实的NSCLC标本115例，肺良性病变组织19例(5例肺结核、4例支气管扩张、6例肺大疱、4例炎性假瘤)。115例NSCLC病人详细病理特征见表 1，分期采用国际抗癌联盟1997年修订的肺癌分期标准。全部病例术前均未作过化疗或放疗；73.9%的病人术后予以铂类为基础的2-4个疗程的化疗。纳入的病例要求切缘阴性，术后生存3个月以上，术后5年内肺癌是其唯一死因。本研究中病人平均生存时间为22个月(3个月-82个月)，随访的截止日期为2009年3月21日。

**1:** 115例非小细胞肺癌病人临床特点 Characteristics of 115 patients with NSCLC

Characteristics	*n*	%
Sex		
Male	87	75.7
Female	28	24.3
Age		
< 60	44	38.3
≥60	71	61.7
Smoking		
Non-smoker	47	40.9
Smoker	68	59.1
Site		
Left	61	53
Right	54	47
Size of tumor		
≤3 cm	32	27.8
> 3 cm	83	72.2
T-stage		
T1+T2	89	77.4
T3+T4	26	22.6
Histology		
Squamous cell carcinoma	40	34.8
Adenocarcinoma	63	54.8
Others	12	10.4
N-stage		
N0	42	36.5
N1+N2	73	63.5
pTNM stage		
Ⅰ	36	31.3
Ⅱ	27	23.5
Ⅲ	41	35.7
Ⅳ	11	9.6
BAC		
Yes	13	11.3
No	102	88.7
Grade		
Poor	67	58.3
Well/Moderate	48	41.7
Wnt1		
(-)	43	37.4
(+)	72	62.6

### 1.2 方法

采用免疫组化Envision方法^[4]^，取病理科石蜡包埋的组织，切片成4 μm，经常规脱蜡至水后，EDTA液(北京中杉金桥生物技术有限公司)高温抗原修复，PBS冲洗1次(1 min)，每张切片滴加50 μL的一抗(兔抗人Wnt1单克隆抗体，英国Abcam公司，稀释浓度1:200)，4 ℃冰箱孵育过夜，PBS冲洗3次，每次1 min，每张切片滴加50 μL的二抗(二步法抗兔/鼠通用型免疫组化试剂盒，丹麦Dako公司)，室温孵育30 min，PBS冲洗3次，每次1 min，DAB显色，苏木素复染，脱水，二甲苯透明，中性树胶封片。阴性对照为PBS代替一抗。

### 1.3 结果判定

以PBS代替一抗设定为阴性空白对照，同时用已知阳性标本作为阳性对照。Wnt1蛋白主要位于细胞浆中，细胞浆显示棕色提示Wnt1阳性表达。Wnt1阳性判断标准参考文献^[5, 6]^，随机选取5个高倍镜视野，每个视野计数100个细胞，计算阳性细胞数占总细胞数的百分比，取5个视野的算术平均值。截断值取50%，即≥50为阳性， < 50%为阴性。

### 1.4 统计处理

采用SPSS 13.0软件进行分析处理，Wnt1表达阳性与阴性病例的比较采用χ^2^检验，生存分析的比较用*Kaplan-Meier*生存曲线，组间比较采用*Log-rank*检验，影响生存期的相关因素分析采用*Cox*多因素回归分析法，所有检验和*P*值均为双侧，*P* < 0.05为差异有统计学意义。

## 2 结果

### 2.1 Wnt1蛋白在非小细胞肺癌中的表达

免疫组化的结果显示Wnt1在NSCLC中的表达位于细胞浆中(图 1)，在NSCLC和肺良性病变组织的表达的阳性率分别为62.6%、31.6%。Wnt1蛋白在NSCLC组织中的表达显著高于对照组(χ^2^=4.474, *P*=0.034)(表 2)。

**1:**
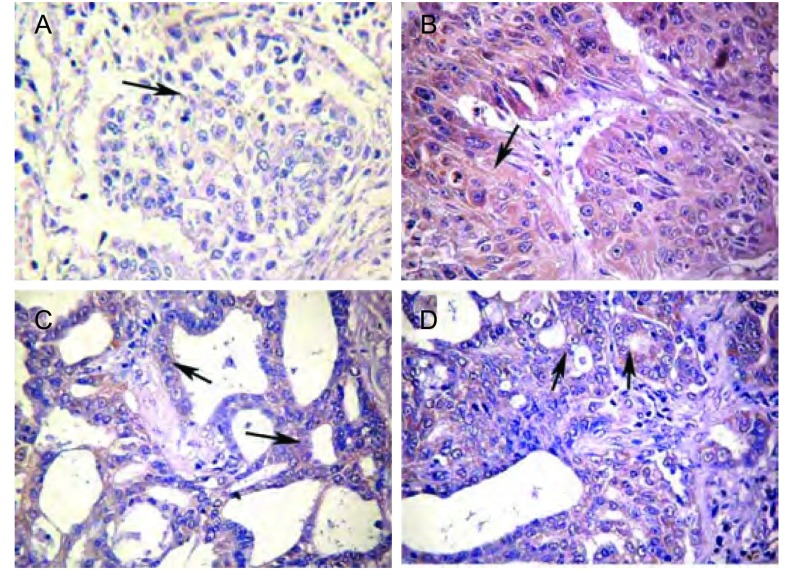
Wnt1在非小细胞肺癌中的表达情况（DAB，×400）。A：Wnt1在鳞癌组织中的阴性表达；B：Wnt1在鳞癌组织中的阳性表达；C、D：Wnt1在腺癌中的阳性表达。 Expression of Wnt1 in NSCLC (DAB, ×400). A: Negative expression of Wnt1 in squamous cell carcinoma; B: Positive expression of Wnt1 in squamous cell carcinoma; C, D: Positive expression of Wnt1 in adenocarcinoma.

**2:** Wnt1蛋白在NSCLC和良性病变组织中的表达 Expression of Wnt1 in NSCLC and benign lesion

Tissue	Wnt1		Positive (%)	*X*^2^	*P*
	(+)	(-)			
NSCLC	72	43	62.6%	4.474	0.034
Benign lesion	6	13	31.6%		

### 2.2 Wnt1蛋白在非小细胞肺癌中的表达和临床病理特征的关系

Wnt1蛋白在NSCLC中的表达与性别、年龄、吸烟、肿瘤部位、肿瘤大小、T分期、病理类型、淋巴结分期、pTNM分期、是否为BAC和肿瘤细胞的分化无明显相关性(表 3)。

**3:** NSCLC组织中Wnt蛋白表达与临床病理参数特征的关系 Relationship between the expression of Wnt1 and clinicopathological characteristics of NSCLC patients

Characteristics	*n*	WNT1		*X*^2^	*P*
		(-)	(+)		
Sex				1.291	0.256
Male	87	30	57		
Fmale	48	13	15		
Age				3.252	0.071
< 60	44	21	23		
≥60	71	22	49		
Smoking				1.804	0.179
Non-smoker	47	21	26		
Smoker	68	22	46		
Site				1.176	0.278
Left	61	20	41		
right	54	23	31		
Size of tumor				0.198	0.656
≤3 cm	32	13	19		
> 3 cm	83	30	53		
T-stage				0.016	0.898
T1+T2	89	33	56		
T3+T4	26	10	16		
Histology				1.237	0.539
Squamous cell carcinoma	40	13	27		
Adenocarcinoma	63	24	39		
Others	12	6	6		
N-stage				2.957	0.086
N0	42	20	22		
N1+N2	73	23	50		
pTNM stage				2.342	0.505
Ⅰ	36	17	19		
Ⅱ	27	9	18		
Ⅲ	41	14	27		
Ⅳ	11	3	8		
BAC				0.007	0.933
Yes	13	5	8		
No	102	38	64		
Grade				< 0.001	0.984
Poor	48	18	30		
Well/Moderate	67	25	42		

### 2.3 Wnt1蛋白的表达与非小细胞肺癌预后的关系

115例NSCLC患者从手术日期开始随访，至患者死亡结束，未死亡的至2009年3月21日截止。用*Kaplan-Meier*生存分析，*Log-rank*检验，Wnt1蛋白阳性表达组与阴性表达组在生存上有统计学差异(*P*=0.03)(图 2)；对性别、年龄、吸烟、肿瘤部位、肿瘤大小、T分期、病理类型、淋巴结转移、是否为BAC、肿瘤细胞的分化、pTNM分期、Wnt1蛋白等12个因素进行*Cox*模型多因素分析，结果表明，Wnt1蛋白和肿瘤分化是影响非小细胞肺癌预后的独立危险因素，Wnt1蛋白阳性的患者预后差(OR=1.834, *P*=0.032)，肿瘤细胞分化越差的患者预后越差(OR=2.931, *P* < 0.001)(表 4)。

**2:**
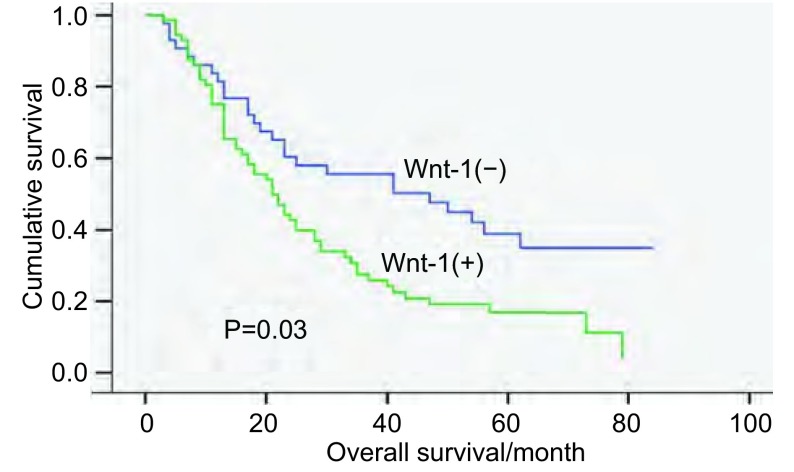
Wnt1蛋白的表达与115例NSCLC患者*Kaplan-Meier*生存分析 *Kaplan-Meier* survival curve of the expression of Wnt1 in 115 patients with NSCLC

**4:** NSCLC预后因素多变量*Cox*回归分析 Multivariate regression analysis in predicting survival of 115 patients with NSCLC

	B	SE	Wald	df	Sig.	Exp(B)	95.0%CI for Exp(B)	
							Lower	Upper
Sex	-0.372	0.344	1.171	1	0.279	0.689	0.351	1.353
Age	-0.535	0.293	3.341	1	0.068	0.585	0.330	1.039
Smoking	-0.328	0.319	1.053	1	0.305	0.721	0.385	1.347
Size of tumor	-0.308	0.292	1.114	1	0.291	0.735	0.414	1.303
Site	-0.435	0.255	2.919	1	0.088	0.647	0.393	1.066
Histology	-0.034	0.225	0.023	1	0.879	0.966	0.622	1.501
BAC	-0.196	0.432	0.205	1	0.650	0.822	0.353	1.916
T-stage	0.287	0.371	0.601	1	0.438	1.333	0.645	2.756
N-stage	0.126	0.436	0.083	1	0.773	1.134	0.483	2.665
pTNM	0.367	0.229	2.557	1	0.110	1.443	0.921	2.261
Grade	1.075	0.259	17.253	1	0.000	2.931	1.765	4.869
Wnt1	0.607	0.283	4.595	1	0.032	1.834	1.053	3.194
B for the partial regression coefficient; SE is standard error of partial regression coefficients; weld test statistic for the overall partial regression coefficients and 0 whether there were significant differences; df degrees of freedom; Sig that *P* value; Exp (B) for the relative risk; 95% CI, confidence interval.								

## 3 讨论

参考文献Wnt信号转导通路可分为经典通路和非经典通路两类，而目前认为经典通路调控细胞增殖和活化，与肿瘤的发生发展密切相关^[7]^。Wnt蛋白作用于细胞膜上的受体，信号传入细胞后，经散乱蛋白传至糖原合成酶激酶-3β(glycogen synthase kinase-3β, GSK-3β)、β连环蛋白、APC蛋白和轴蛋白，形成多聚蛋白复合体。GSK-3β的磷酸化作用引起β连环蛋白降解，使后者在细胞质内的游离量保持在较低水平；当GSK-3β被Wnt信号抑制后，β连环蛋白的降解随即被中断，使细胞内游离的β连环蛋白含量增加，并进入核内，作用于T细胞因子/淋巴样增强因子(T cell factor/lymphocyte enhancer factor, TCF/LEF)，并形成β连环蛋白-TCF/LEF转录复合体，最终激活Wnt信号的有关靶基因(主要为c-myc和cyclin D1)，调控胚胎发育及细胞生长、分化和凋亡。

已发现Wnt1与许多肿瘤有关，包括结直肠癌、食管癌、胃癌、胰腺癌、头颈部肿瘤、黑素瘤、肉瘤、白血病、基底细胞癌、肺癌、间皮瘤^[8-10]^。Wnt1蛋白在NSCLC组织中的表达目前报道较少，国内目前只有两篇小样本(分别为60例、63例)文献报道^[11, 12]^，这两篇报道均未阐述Wnt1表达与预后的关系，国外同一所医院的Na-kashima、Huang等^[6, 13]^报道Wnt1蛋白在NSCLC组织中表达与预后相关，并可作为独立的预后因素。本研究通过免疫组化的方法检测NSCLC组织内Wnt1蛋白的表达情况及与临床病理特征的相关性。结果显示Wnt1蛋白在NSCLC中的阳性表达显著高于肺良性病变组织，但与临床病理特征无明显的相关性。本实验通过随访获得患者的生存期，用*Kaplan-Meier*生存分析、*Log-rank*检验提示Wnt1阳性表达的NSCLC患者预后较差，*Cox*模型多因素分析结果表明Wnt1蛋白是影响NSCLC预后的独立危险因素，与Nakashima、Huang等^[6, 13]^的研究结果一致。提示Wnt1蛋白的阳性表达可以作为判断NSCLC预后的独立指标之一。Nakashima、Huang等^[6, 13]^同时通过研究得出Wnt1的表达与其下游的Ki-67、c-Myc、Cylin D1、MMP-7、VEGF-A有关，而上述因子主要与肿瘤的增殖及肿瘤新生血管形成有关，故这是Wnt1的可能作用机制。本文是国内第一篇研究Wnt1与NSCLC预后的文献，同时本文还研究了Wnt1的表达与临床病理特征的相关性，其结果与两篇国外报道一致，而国内的两篇文献报道其阳性表达与部分临床病理特征相关，目前国内外关于Wnt1预后的文献报道较少，其确切的机制及与临床病理特征的相关性有待进一步探讨。

分子靶向治疗的发展开创了NSCLC治疗的新领域，目前已被NCCN推荐作为二线药物及部分患者的一线药物，寻找作用于新的靶点的药物是近年来研究的热点。抑制肺癌组织中Wnt信号异常表达可以诱导细胞凋亡、抑制肿瘤增殖，利用该通路中特异信号分子及其下游效应物可以实施肿瘤靶向性治疗策略，通过基因选择性杀伤恶性肿瘤细胞。目前，已发现作用于Wnt信号通路的拮抗剂有DKK(Dickkopf)家族、分泌型frizzled相关蛋白(secreted frizzled-related protein family, sFRP)、Wnt抑制因子-1(Wnt inhibitory factor-1, WIF-1)^[14, 15]^和内皮抑素^[16]^。随着对Wnt信号通路及其各成员研究的不断深入，我们对Wnt信号通路在肿瘤发生发展中的作用将会进一步明确，预计针对Wnt信号通路不同位点的特异性靶向治疗药物将得到开发。

虽然对Wnt通路的研究取得许多突破，但仍有很多方面有待进一步研究，如对该通路中的一些未知因子的探索；进一步研究Wnt信号转导通路中各基因及表达蛋白的互相作用，从而更深层次阐明肿瘤的发生机制；在单一细胞中Wnt信号转导通路研究的基础上，进一步分析与其他通路之间的相互联系；将Wnt信号转导通路作为抗肿瘤治疗起点，研制抗肿瘤药物应用于临床有待深入研究。
